# Transcriptomic profiles of *Clostridium ljungdahlii* during lithotrophic growth with syngas or H_2_ and CO_2_ compared to organotrophic growth with fructose

**DOI:** 10.1038/s41598-017-12712-w

**Published:** 2017-10-13

**Authors:** Muktak Aklujkar, Ching Leang, Pravin M. Shrestha, Minita Shrestha, Derek R. Lovley

**Affiliations:** 1 University of Massachusetts, Amherst, MA 01003 USA; 2 University of Nevada, Reno, NV 89557–0352 USA; 3 LanzaTech, Skokie, 60077 IL USA; 4 Energy Biosciences Institute, University of California, Berkeley, CA 94720 USA; 5 Earth and Environmental Sciences, Lawrence Berkeley National Laboratory, Berkeley, CA 94720 USA

## Abstract

*Clostridium ljungdahlii* derives energy by lithotrophic and organotrophic acetogenesis. *C*. *ljungdahlii* was grown organotrophically with fructose and also lithotrophically, either with syngas - a gas mixture containing hydrogen (H_2_), carbon dioxide (CO_2_), and carbon monoxide (CO), or with H_2_ and CO_2_. Gene expression was compared quantitatively by microarrays using RNA extracted from all three conditions. Gene expression with fructose and with H_2_/CO_2_ was compared by RNA-Seq. Upregulated genes with both syngas and H_2_/CO_2_ (compared to fructose) point to the urea cycle, uptake and degradation of peptides and amino acids, response to sulfur starvation, potentially NADPH-producing pathways involving *(S)*-malate and ornithine, quorum sensing, sporulation, and cell wall remodeling, suggesting a global and multicellular response to lithotrophic conditions. With syngas, the upregulated *(R)*-lactate dehydrogenase gene represents a route of electron transfer from ferredoxin to NAD. With H_2_/CO_2_, flavodoxin and histidine biosynthesis genes were upregulated. Downregulated genes corresponded to an intracytoplasmic microcompartment for disposal of methylglyoxal, a toxic byproduct of glycolysis, as 1-propanol. Several cytoplasmic and membrane-associated redox-active protein genes were differentially regulated. The transcriptomic profiles of *C*. *ljungdahlii* in lithotrophic and organotrophic growth modes indicate large-scale physiological and metabolic differences, observations that may guide biofuel and commodity chemical production with this species.

## Introduction

*C*. *ljungdahlii* is capable of lithotrophic growth by the Wood-Ljungdahl pathway with hydrogen as the electron donor, carbon dioxide as the electron acceptor, and acetate as the major end product, with a small amount of acetyl-CoA being assimilated into biomass^1^. *C*. *ljungdahlii* can also utilize syngas, a gas mixture containing H_2_, CO_2_ and CO, as a lithotrophic substrate for the Wood-Ljungdahl pathway^1^. *C*. *ljungdahlii* also performs electrosynthesis, the reduction of CO_2_ to acetate with electrons derived from electrodes^2,3^. The results of genetic modifications to redirect carbon and electron flow to products other than acetate suggest that *C*. *ljungdahlii* may serve as an effective chassis for converting CO_2_ to fuels or other organic commodities^4–8^.

Various carbohydrates and amino acids also support the growth of *C*. *ljungdahlii* ^1^. Under organotrophic conditions, *C*. *ljungdahlii* continues to operate the Wood-Ljungdahl pathway as an energetically favourable means to dispose of electrons from oxidation of the organic substrate. For example, glycolytic fermentation of fructose to lactate yields only two ATP, whereas oxidation of fructose to acetate plus CO_2_ and use of the electrons to fix CO_2_ into acetate by the Wood-Ljungdahl pathway yields four ATP^9^ minus an estimated 0.14 ATP for reverse electron transport (using reactions described below and assuming that hydrolysis of 3 ATP pumps 11 protons)^10^.

The discovery of bifurcated electron transfer^11^ has led to the realization that metabolic pathways that were thought to be strictly fermentative (lacking electron transport, with substrate-level phosphorylation only) may conserve energy by coupling exergonic redox reactions to electron transfer from NADH to ferredoxin (Fd_ox_). This energy is converted into transmembrane potential with electron transport from ferredoxin (Fd_rd_) to NAD by the Rnf complex, which pumps protons or sodium ions.

**Figure 1:**
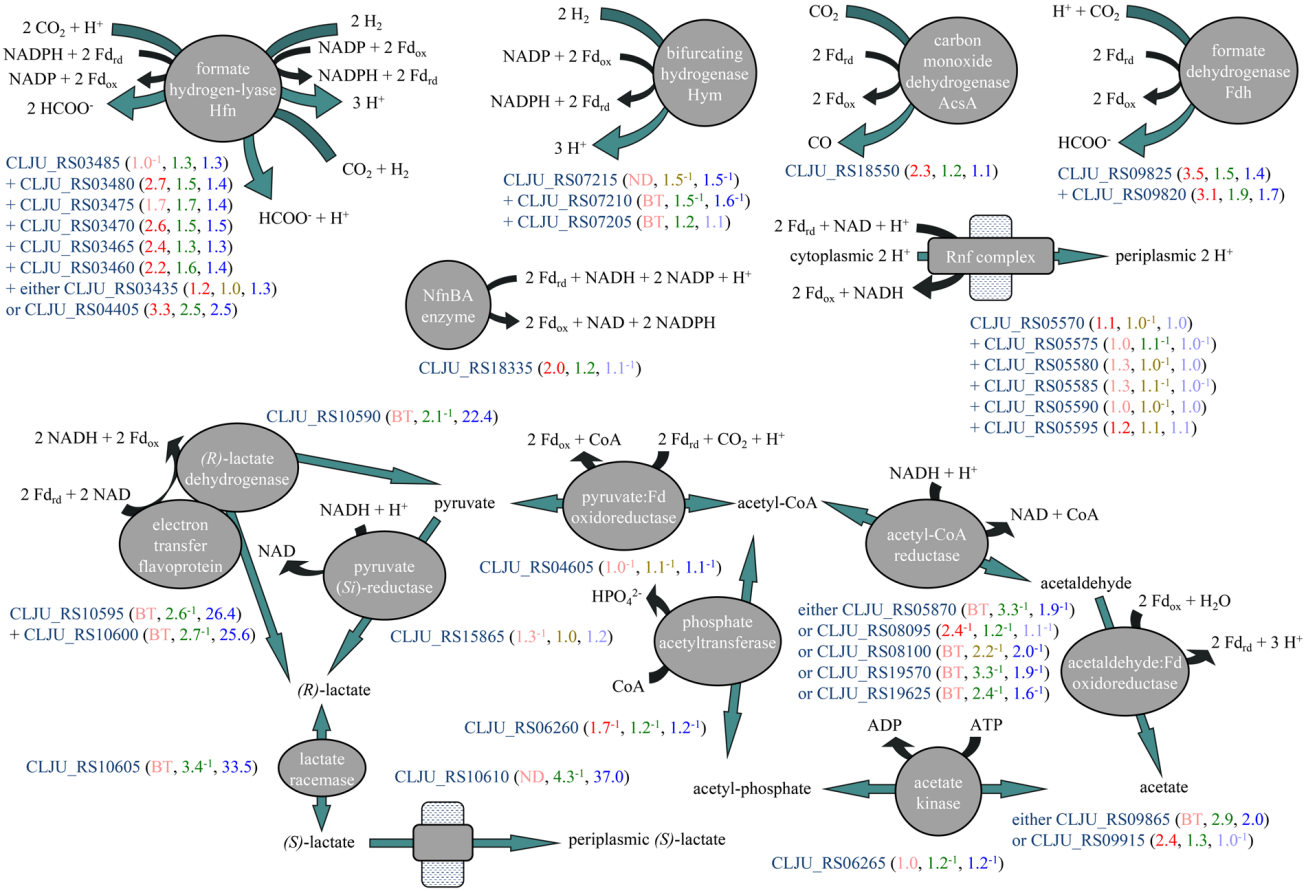
Reactions that distribute electrons to NAD, NADP and ferredoxin (Fd_ox_) in *C*. *ljungdahlii* growing lithotrophically. Blue-green arrows stand for reactions and black arrows for oxidation/reduction of electron carriers, consumption/production of ATP, and similar effects. Fold changes in the transcript level of each gene are indicated according to RNA-Seq of H_2_/CO_2_-grown *versus* fructose-grown cells (red; statistically insignificant changes in coral; BT = below threshold for detection of differential expression, log_2_ RPKM < 5; ND = not detected), microarray analysis of H_2_/CO_2_-grown *versus* fructose-grown cells (green; insignificant changes in olive), and microarray analysis of syngas-grown *versus* fructose-grown cells (blue; insignificant changes in light-blue). Fold changes with the exponent −1 signify downregulation. There are two putative H_2_-oxidizing enzymes: formate hydrogen-lyase Hfn and bifurcating hydrogenase Hym; and two formate-producing enzymes: Hfn and formate dehydrogenase Fdh; the AcsA subunit of acetyl-CoA synthase is carbon monoxide dehydrogenase. The proton-pumping Rnf complex and the NADH-dependent ferredoxin:NADP oxidoreductase Nfn conserve energy as they exchange reduced ferredoxin (Fd_rd_), NADH and NADPH. Inhibition of Rnf by a strong proton gradient would explain the dramatic upregulation of enzymes of *(R)*-lactate cycling that do not conserve energy from the exchange of two Fd_rd_ for NADH during growth with syngas. During growth with H_2_/CO_2_, upregulation of aldehyde:ferredoxin oxidoreductases may maintain a reduced ferredoxin pool.

The Wood-Ljungdahl pathway consumes one ATP to ligate formate with tetrahydrofolate (THF) and yields one ATP when acetyl-CoA is converted via acetyl-phosphate into acetate. Therefore, its net energy yield depends on the differences in redox potential between the substrates of organotrophy (e.g. fructose) or lithotrophy (e.g. H_2_ or CO) and the electron carriers oxidized by four oxidoreductases of the Wood-Ljungdahl pathway. The first oxidoreductase is either formate dehydrogenase (Fig. 1), predicted to accept two electrons from the one-electron carrier Fd_rd_ through a ferredoxin-like iron-sulfur cluster-binding subunit, or a formate hydrogen-lyase that accepts one electron from Fd_rd_ and one from NADPH^12,13^. The second oxidoreductase is NADPH-dependent methenyl-THF reductase. The third is methylene-THF reductase, for which the electron carrier is unknown. It is hypothesized to perform bifurcated electron transfer by oxidizing two NADH and reducing two Fd_ox_ to Fd_rd_ as it reduces methylene-THF to methyl-THF^9^. The fourth oxidoreductase is the carbon monoxide dehydrogenase subunit of acetyl-CoA synthase (Fig. 1), which is predicted to require two electrons at the low redox potential of Fd_rd_. Thus, the exchange of electrons among Fd_rd_, NADH, and NADPH is fundamental to the operation of the Wood-Ljungdahl pathway regardless of the mode of growth. Two enzymes central to this exchange are the proton-translocating Rnf complex^14^, which reduces NAD with two Fd_rd_ (Fig. 1), and the Nfn enzyme^15^ that transfers four electrons from one NADH and two Fd_rd_ to two NADP (Fig. 1).

During organotrophic growth by oxidation of fructose to acetyl-CoA, the glycolytic enzymes glyceraldehyde-3-phosphate dehydrogenase and pyruvate:ferredoxin oxidoreductase donate equal numbers of electrons to NAD and Fd_ox_. This may allow the cell to derive all of its NADPH from the Nfn enzyme. In contrast, lithotrophic substrates such as H_2_ and syngas will distribute electrons unevenly to NAD, NADP and Fd_ox_. Nfn-independent pathways may then be required to provide NADH or NADPH. Oxidation of carbon monoxide, the energetically preferable lower-redox-potential electron donor present in syngas, may be attributed to the carbon monoxide dehydrogenase subunit of acetyl-CoA synthase, which reduces only Fd_ox_ (Fig. 1). Analysis of the *C*. *ljungdahlii* genome predicts that H_2_ is oxidized by two enzyme complexes that bind NADP at soluble ligand-binding beta-grasp (SLBB) domains^16^: a bifurcating hydrogenase called Hym that couples electron transfer from H_2_ to the higher redox potential of NADP and the lower redox potential of Fd_ox_ (Fig. 1) and a formate hydrogen-lyase called Hfn - or Hyt in *Clostridium autoethanogenum* ^13^ - that oxidizes H_2_ to reduce CO_2_ to formate and is also capable of bifurcating electrons from H_2_ to NADP and to Fd_ox_ (Fig. 1). Two of the three Fd_rd_ produced from 3 H_2_ by Hym or Hfn would be consumed by carbon monoxide dehydrogenase (Fig. 1) and one NADPH would be consumed by methenyl-THF reductase; therefore, *C*. *ljungdahlii* growing under lithotrophic conditions with H_2_ would perform electron transport through Rnf to convert the remaining 0.5 NADPH and one Fd_rd_ into NADH or another electron carrier for methylene-THF reductase. It is not known how an excess of Fd_rd_ or an excess of NADPH may impact the Wood-Ljungdahl pathway.

Previously published transcriptomic studies of *C*. *ljungdahlii* evaluated responses to growth with CO/CO_2_ or fructose^17^, growth with H_2_/CO_2_ or fructose and with NH_4_^+^ or NO_3_^−^ as the nitrogen source^18^, O_2_ stress^19^, and NaCl stress^20^ using RNA-Seq. In this study, the transcriptomic profiles of *C*. *ljungdahlii* grown lithotrophically with either syngas or H_2_/CO_2_ and organotrophically with fructose were compared using microarrays, and gene expression with H_2_/CO_2_ and with fructose was also compared using RNA-Seq. This approach allowed patterns of differential regulation to be identified as common to the two previously untested lithotrophic growth conditions compared to fructose, such as upregulation of urea cycle genes and downregulation of microcompartment genes, while other genes were upregulated only with syngas, such as *(R)*-lactate dehydrogenase, or only with H_2_/CO_2_, such as histidine biosynthesis genes. Many redox-active protein genes were also identified as differentially regulated.

## Results and Discussion

### Comparison of RNA-Seq and microarray datasets

The total number of protein-coding genes identified in the genome of *C*. *ljungdahlii* is 4074, including genes that appear frameshifted in homopolymeric tracts, but excluding gene fragments. There are eight sets of two genes and five sets of five genes with ~100% DNA sequence identity, for which gene expression had to be measured collectively, and five sets of two genes with 97–99% DNA sequence identity, which shared some microarray probes. The microarray detected 4019 gene transcripts and RNA-Seq detected 3795 gene transcripts in total. Five gene transcripts without microarray probes were detected by RNA-Seq, but not differentially expressed. Of the 229 gene transcripts that were detected only by microarray, 84 genes showed significant differential expression (*p* ≤ 0.05). The number of genes differentially expressed (*p* ≤ 0.05) according to at least one method was 3341, of which 66 genes were disqualified because they were below the threshold of significance (median reads assigned per kilobase of target per million mapped reads (RPKM) ≥5) for RNA-Seq and did not meet the *p* value criterion for the microarray. Differentially expressed genes were ranked according to their largest significant fold change (Supplementary Table S1).

Many differentially expressed genes encode predicted transcriptional regulators or other genes that are expected to affect the expression of other proteins (Supplementary Table S2).

In the discussion below, genes are described as upregulated if expression was significantly (*p* ≤ 0.05) greater for lithotrophic growth with either syngas or H_2_/CO_2_ (by microarray analysis and/or RNA-Seq) compared to fructose, and as downregulated if expression was significantly (*p* < 0.05) lesser for a lithotrophic growth condition than for organotrophic growth with fructose. Genes for which the fold change was tenfold or larger are termed “strongly upregulated” or “strongly downregulated”.

### *(S)*-malate uptake and oxidation during lithotrophic growth

**Figure 2:**
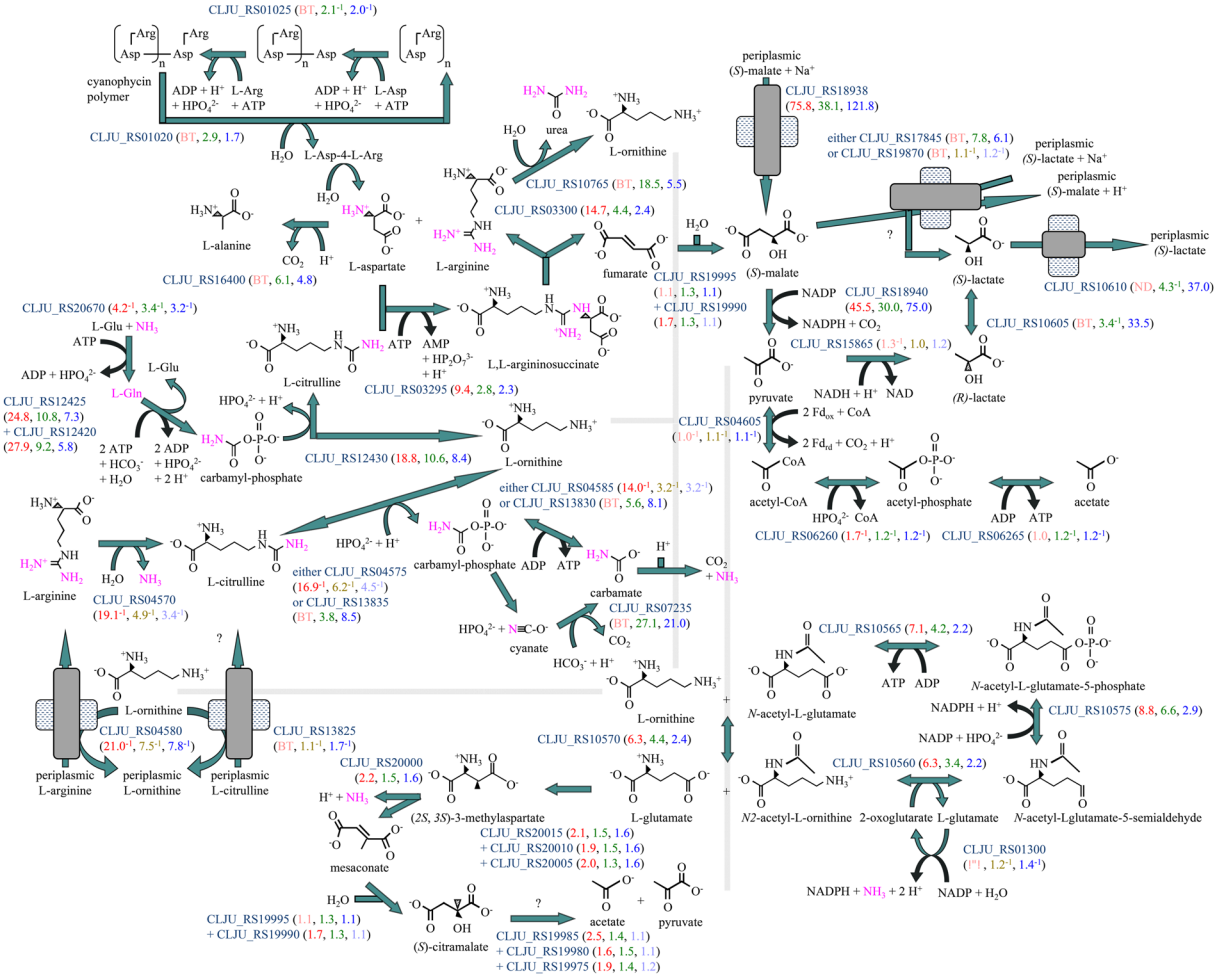
Amino acid degradation pathways that may be operated by lithotrophic *C*. *ljungdahlii*, including the urea cycle and uptake and oxidation of *(S)*-malate. Fold changes are indicated as in Fig. 1. Nitrogen being excreted is shown in pink. Arginine, derived either from the storage polymer cyanophycin or from yeast extract in the medium, is split into urea plus ornithine, which is degraded through glutamate, releasing ammonia. To prevent an increase in pH, ammonia is recaptured by glutamine synthetase and converted into carbamyl-phosphate, which reacts with ornithine and the amino group of aspartate in the urea cycle. The fumarate released from aspartate is degraded through *(S)*-malate, with *(S)*-lactate possibly excreted as an electron sink and then recaptured in exchange for *(S)*-malic acid, which is later recaptured; this transient removal of intermediates may ensure that amino acid degradation is sustained and that ammonia is sequestered as urea so that the pH does not rise. Concomitant upregulation of genes for citrulline fermentation, but not uptake of arginine/citrulline or fermentation of arginine through citrulline, suggests that citrulline is diverted from the urea cycle to release carbamyl-phosphate, from which ATP can be recovered and ammonia is released. Upregulation of cyanate lyase implies a high level of carbamyl-phosphate that spontaneously rearranges into cyanate and must be detoxified.

The two most upregulated genes during lithotrophic growth with syngas (*maeN* CLJU_RS18938, *ytsJ-2* CLJU_RS18940) encode a protein with 55% sequence identity to the characterized sodium/*(S)*-malate symporter of *Bacillus subtilis* ^21^ and one of the four putative malic enzymes of *C*. *ljungdahlii* with 64% protein sequence identity to a major NADP-reducing enzyme that oxidatively decarboxylates *(S)*-malate to pyruvate in *B*. *subtilis* ^22,23^. During lithotrophic growth with H_2_/CO_2_, these two genes were the fourth and sixth most upregulated genes according to the microarray and the first and fourth most upregulated genes according to RNA-Seq (Fig. 2). Genes for the *(S)*-malate sensor kinase and response regulator (*malK* CLJU_RS18950, *malR* CLJU_RS18945) were also upregulated with syngas and with H_2_/CO_2_ (Supplementary Table S2). All of this indicates that lithotrophically grown *C*. *ljungdahlii* attempts to take up and oxidize *(S)*-malate even though the growth medium contains no organic carbon other than 0.1% yeast extract, vitamins, and nitrilotriacetate. Perhaps *C*. *ljungdahlii* is accustomed to encounter *(S)*-malate along with lithotrophic substrates in its natural environment; alternatively, it may be responding gratuitously to a molecule that mimics *(S)*-malate (one possibility being the tricarboxylate molecule nitrilotriacetate, which is present in the growth medium), or it may be responding to *(S)*-malate that it has previously excreted.

*C*. *ljungdahlii* possesses two homologs of the *(S)*-malic acid/sodium *(S)*-lactate antiporter of *B*. *subtilis* ^21^. One homolog (CLJU_RS19870; 32% protein sequence identity) was not differentially regulated with syngas or H_2_/CO_2_ *versus* fructose, although it was the most upregulated gene under NaCl stress^20^ and the tenth most upregulated gene with CO/CO_2_ *versus* fructose^17^. The other homolog (CLJU_RS17845; 22% protein sequence identity) was upregulated with both syngas and H_2_/CO_2_ (Fig. 2). If *C*. *ljungdahlii* uses this antiporter to import *(S)*-lactate or a similar organic acid and export *(S)*-malate, the sodium/*(S)*-malate symporter’s function could be to recapture *(S)*-malate. The medium does not contain *(S)*-lactate, but it may be transiently produced by degradation of the amino acids in yeast extract or cyanophycin, a storage polymer of aspartate and arginine (Fig. 2). This idea is consistent with upregulation of genes for urea cycle enzymes (Fig. 2) by which arginine and aspartate are degraded to ornithine, urea and fumarate, which can be hydrated to *(S)*-malate. Oxidative decarboxylation of *(S)*-malate to pyruvate and of pyruvate to acetyl-CoA produces NADPH and two Fd_rd_, which can be reoxidized by the Wood-Ljungdahl pathway or exchanged for NADH by the Nfn and Rnf enzymes (Fig. 1), generating a proton gradient. Conversion of acetyl-CoA into acetate either generates ATP or exchanges NADH for two Fd_rd_ (Fig. 1). Therefore, either a high ATP:ADP ratio or a high Fd_rd_:Fd_ox_ ratio could cause pyruvate to accumulate, in which case reduction of pyruvate with NADH to *(R)*-lactate, racemization to *(S)*-lactate, and excretion would allow sustained amino acid degradation. Subsequent recapture of the electron sink, *(S)*-lactate, by exchange with *(S)*-malic acid could serve three purposes: to recover electrons; to acidify the medium to counteract the release of ammonia from amino acids; and to promote sequestration of ammonia by the urea cycle by removing one product, fumarate (Fig. 2). At a later stage, when amino acids are unavailable to supplement lithotrophic growth, *(S)*-malate could be recaptured and oxidized.

### Upregulation of cytoplasmic redox-active protein genes

Three of the twenty-five most upregulated genes with H_2_/CO_2_ are predicted to be co-transcribed and encode a transcriptional regulator, a flavodoxin, and a protein of unknown function (Supplementary Table S3). Not one of these genes was differentially regulated with syngas, suggesting that their products function only in lithotrophic growth with H_2_/CO_2_. (Previously, the flavodoxin was not detected in a syngas proteomic sample^24^). Several other genes predicted to encode cytoplasmic redox-active proteins were upregulated with H_2_/CO_2_ and/or syngas (Supplementary Table S3). It might be valuable to study whether their functions, such as reduction of disulfide bonds and detoxification of reactive oxygen species, are more important for lithotrophic growth.

### Upregulation of membrane-associated redox protein genes

*C*. *ljungdahlii* does not possess genes for biosynthesis of heme or the redox-active portion of any known quinone^9^, and therefore appears to lack electron transport pathways except for the Rnf complex. However, when *C*. *ljungdahlii* consumes an electrical current to perform electrosynthetic carbon fixation^3^, electrons must traverse the membrane to reach cytoplasmic electron carriers, perhaps through other membrane-associated redox proteins. One such protein complex may be the product of an operon that was upregulated with syngas and with H_2_/CO_2_ (Supplementary Table S4), encoding a membrane protein of the DsbD superfamily that may form redox-active disulfide bonds (CLJU_RS13020), an iron-sulfur cluster-binding membrane protein (CLJU_RS13025), a part of which resembles the ubiquinol-oxidizing NapH subunit of the periplasmic nitrate reductase of *E*. *coli* ^25^, and a periplasmic protein (CLJU_RS13030). The substrate specificity of this putative oxidoreductase is an interesting topic for future research.

Several other genes for redox-active proteins with predicted membrane associations were upregulated with syngas and/or H_2_/CO_2_ (Supplementary Table S4). Notable among them is the first of two tandem gene sets that each encode homologs of four proteins. The first protein is a (molybdopterin cytosine dinucleotide)-oxothiomolybdenum-binding aldehyde oxidoreductase (CLJU_RS11830, CLJU_RS11870). The second protein is a fused FAD-binding pyridine nucleotide-disulfide oxidoreductase and CCG domain pair iron-sulfur cluster-binding oxidoreductase (CLJU_RS11825, CLJU_RS11865), which may reduce or oxidize a membrane-bound electron carrier through the CCG domain pair by analogy with quinone/phenazine oxidoreductases^26,27^. The third protein has four conserved cysteine residues (CLJU_RS11820, CLJU_RS11860). The fourth protein is of the C_GCAxxG_C_C family (CLJU_RS11815, CLJU_RS11850). Both proteins could be redox-active. The nature of the redox reactions carried out by these gene products should be investigated for their possible relevance to electron transport.

### Lactate oxidation as a ferredoxin reoxidation strategy during growth with syngas

The fourth and fifth most upregulated genes during growth with syngas (*lctP* CLJU_RS10610 and *larA-3* CLJU_RS10605, Fig. 1) encode proteins with 49% sequence identity to the predicted *(S)*-lactate transporter of *Streptococcus iniae* ^28^ and 63% sequence identity to the characterized lactate racemase of *Lactobacillus plantarum* ^29^, respectively. The ninth, tenth and thirteenth most upregulated genes (*lutJ* CLJU_RS10595, *lutI* CLJU_RS10600 and *lutK* CLJU_RS10590, Fig. 1) encode an electron transfer flavoprotein and a candidate *(R)*-lactate dehydrogenase with 28% protein sequence identity to an *(R)*-lactate-oxidizing enzyme of *E*. *coli* ^30^. These genes are predicted to be in an operon, possibly including the twelfth most upregulated gene (CLJU_RS10585, 23.5-fold) that encodes a protein of the hemerythrin-like superfamily. These six genes were only upregulated with syngas (as with CO/CO_2_^17^); with H_2_/CO_2_, they were either downregulated or not differentially regulated (Fig. 1). Their strong upregulation with syngas suggests that oxidation of *(R)*-lactate to pyruvate with NAD, coupled to electron transfer from two Fd_rd_ to NAD^31^, may be a particularly important reaction for *C*. *ljungdahlii* when the electron donor is CO from syngas (Fig. 1).

Carbon monoxide, the energetically preferable electron donor in syngas, is also an intermediate of the Wood-Ljungdahl pathway, produced within acetyl-CoA synthase by the reduction of CO_2_ with two Fd_rd_. Of the three genes of *C*. *ljungdahlii* predicted to encode catalytic subunits of carbon monoxide dehydrogenases, two (*cooS-1* CLJU_RS04490, *cooS-2* CLJU_RS08800) were downregulated with syngas (Supplementary Table S1; as with CO/CO_2_^17^, although the CooS-1 protein was abundant in a proteomic sample^24^) and therefore unlikely to contribute to oxidation of CO. The third gene, for which transcripts were most abundant (*acsA* CLJU_RS18550), is located within the Wood-Ljungdahl pathway gene cluster and presumably functions both to provide CO to acetyl-CoA synthase and to oxidize CO with electron transfer to two Fd_ox_ (Fig. 1). Thus, it is likely that when CO is the electron donor, all electrons pass to Fd_ox_ and must be transferred to NAD and NADP for use in the methyl branch of the Wood-Ljungdahl pathway. The Rnf complex catalyzes electron transport from two Fd_rd_ to NAD (Fig. 1), but only to the extent that it can pump protons against the gradient. When the proton gradient is too strong, another route may be required for electrons from two Fd_rd_ to reach NAD. An electron transfer flavoprotein can provide this route by coupling the exergonic oxidation of two Fd_rd_ by NAD with an endergonic reaction such as oxidation of *(R)*-lactate to pyruvate with reduction of NAD^31^.

*C*. *ljungdahlii* has a putative source for endogenous *(R)*-lactate: an NADH-dependent pyruvate (*Si*)-reductase LdhA, with 100% protein sequence identity to the characterized enzyme of *Clostridium autoethanogenum* ^32^, encoded by a gene that is not differentially expressed (*ldhA* CLJU_RS15865). With no obvious mechanism to conserve energy from this exergonic reaction, the LdhA enzyme may function irreversibly, driving a cycle of pyruvate reduction and *(R)*-lactate reoxidation (Fig. 1). With LdhA oxidizing one NADH and an electron transfer flavoprotein-associated *(R)*-lactate dehydrogenase oxidizing two Fd_rd_ and reducing two NAD, there would be a net irreversible exchange of two Fd_rd_ for one NADH independently of the Rnf complex. We speculate that if this energetically wasteful cycle were abolished, *C*. *ljungdahlii* might conserve all of the available energy from syngas for bioenergy applications.

### Operation of the urea cycle to limit the release of ammonia

The arginase gene (CLJU_RS10765) was upregulated with syngas and with H_2_/CO_2_, suggesting that arginine released by breakdown of the storage polymer cyanophycin may be hydrolyzed to ornithine and urea (Fig. 2). Arginase is the key enzyme of the urea cycle, for which all of the other genes were also upregulated (Fig. 2), suggesting that additional urea is produced from ammonia released by degradation of ornithine and from aspartate released from cyanophycin. The upregulated genes encode one of two isozymes of carbamyl-phosphate synthase and one of three isozymes of ornithine carbamyltransferase, plus argininosuccinate synthetase and argininosuccinate lyase. The argininosuccinate enzymes are encoded by a predicted operon that includes genes for an amino acid uptake ATP-binding cassette (ABC) transporter that were also upregulated with syngas and with H_2_/CO_2_ (Supplementary Table S5). The hypothesis that this transporter enables uptake of exogenous argininosuccinate or cyanophycin units is consistent with the 34–37% sequence identity of its periplasmic amino acid-binding protein to *Escherichia coli* and *B*. *subtilis* proteins for uptake of another side-chain-linked amino acid dimer, cystine^33,34^. The overall gene expression pattern suggests that the enzymes of the urea cycle are abundant in *C*. *ljungdahlii* growing lithotrophically.

The urea cycle expends five ATP to sequester a molecule of ammonia and the amino group of aspartate (Fig. 2), but the estimated energy yield from degradation of ornithine and fumarate to acetate (Fig. 2) with disposal of electrons through the Wood-Ljungdahl pathway is only 3.38 ATP, assuming that 3 ATP are synthesized by passing 11 protons through ATP synthase as in *Clostridium paradoxum* ^10^. Thus, it is energetically prohibitive for *C*. *ljungdahlii* to degrade cyanophycin solely through the urea cycle. Degradation of arginine and aspartate by non-urea-cycle reactions that release ammonia (Fig. 2) is estimated to yield 4.38 ATP. It is probable that *C*. *ljungdahlii* expends some of this energy to operate the urea cycle to mitigate the release of ammonia that raises the cytoplasmic pH. This energetic cost should be considered when choosing nitrogenous supplements for media for large-scale lithotrophic growth of *C*. *ljungdahlii*. Genes for salvaging and detoxification of urea cycle intermediates were also upregulated (Supplementary Discussion S1).

### NADP-reducing degradation of ornithine under lithotrophic conditions

After arginase converts arginine from either cyanophycin or yeast extract into ornithine, *C*. *ljungdahlii* degrades ornithine to glutamate by a reversible pathway, for which all of the genes were upregulated with syngas and with H_2_/CO_2_ (Fig. 2). The alternative interpretation, that these genes were upregulated for biosynthesis of arginine from glutamate, is implausible because the arginase gene was upregulated and the genes for glutamate biosynthesis were downregulated. Furthermore, gene expression patterns were consistent with degradation of glutamate, for which most of the genes were modestly upregulated with syngas and with H_2_/CO_2_ (Fig. 2). Degradation of ornithine to glutamate releases ammonia and produces ATP and two NADPH; degradation of glutamate through pyruvate to acetate releases ammonia and produces ATP and two Fd_rd_ (Fig. 2). Thus, ornithine degradation may augment the pool of NADPH in cells growing with either syngas or H_2_/CO_2_, whereas glutamate degradation, as a source of Fd_rd_, may be beneficial for growth with H_2_/CO_2_.

### Upregulation of histidine biosynthesis and histidine degradation

**Figure 3:**
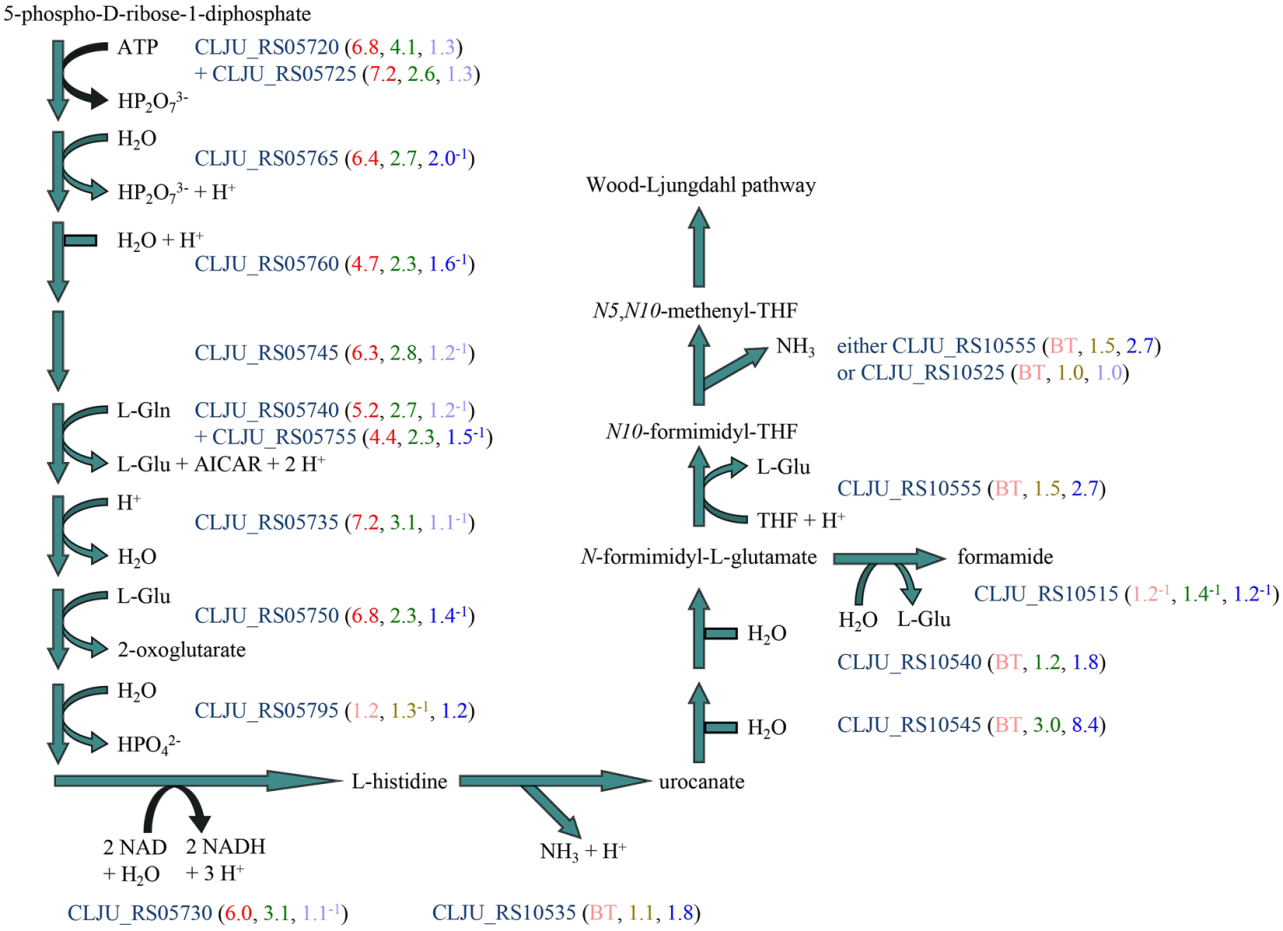
Upregulation of histidine biosynthesis and degradation genes in *C*. *ljungdahlii* growing lithotrophically. Fold changes are indicated as in Fig. 1. Histidine is degraded to glutamate, which is further degraded as in Fig. 2.

The histidine biosynthesis operon was upregulated with H_2_/CO_2_ (as with NaCl stress^20^), but not with syngas (Fig. 3). Only one enzyme of histidine biosynthesis, the putative histidinol-phosphate phosphatase (*hisK* CLJU_RS05795), was not differentially regulated. This pattern suggests that *C*. *ljungdahlii* might need to synthesize proteins with high histidine content specifically to grow lithotrophically with H_2_/CO_2_. To explore this idea, the histidine content of every predicted protein of *C*. *ljungdahlii* was computed. Of those whose histidine content is at least 3.5%, which is two standard deviations (1.1%) above the mean (1.4%), most genes were not upregulated with H_2_/CO_2_ (data not shown). A high-histidine-content protein gene that was upregulated with H_2_/CO_2_ (Supplementary Table S6) encodes a transcriptional regulator (CLJU_RS09505) with a histidine-rich C-terminal extension. Genes upregulated with H_2_/CO_2_ and with syngas encode a protein of unknown function (CLJU_RS11125) between two sets of peptide ABC transporter genes (Supplementary Table S5), for which a paralogous protein (CLJU_RS18470) is encoded on the 3′ side of the Wood-Ljungdahl pathway genes, a repeat-containing protein with two pentahistidine motifs (CLJU_RS07790), and cyanate lyase (CLJU_RS07235; Supplementary Discussion S1). These genes are candidates to be investigated for roles in lithotrophic growth.

Surprisingly, the histidine degradation gene cluster was upregulated with syngas; it encodes a transcriptional regulator (CLJU_RS10550, Supplementary Table S2) and six enzymes that degrade histidine to glutamate (Fig. 3). This pattern suggests that *C*. *ljungdahlii* supplements its lithotrophic metabolism of syngas by degrading histidine, which it may derive from yeast extract. The one-carbon unit in the side-chain of histidine can be either transferred as a formimidyl group to THF to enter the Wood-Ljungdahl pathway (without the expense of one ATP to ligate formate to THF) or hydrolyzed to formamide and oxidized through formate. The gene for *N*-formimidyl-L-glutamate formimidylhydrolase (*hutE* CLJU_RS10515) was the only downregulated gene of histidine degradation, indicating that when syngas is available as an electron donor, *C*. *ljungdahlii* prefers not to oxidize the one-carbon unit and instead uses it to make the methyl group of acetate.

### Upregulation of molybdopterin biosynthesis under lithotrophic conditions

The genes for ferredoxin-dependent formate dehydrogenase, which were modestly upregulated (Fig. 1), are transcribed divergently from two strongly upregulated genes for incorporation of sulfur into molybdopterin, a precursor of the cofactor of formate dehydrogenase: the sulfur carrier protein gene (*moaD-1* CLJU_RS09835) was upregulated with H_2_/CO_2_, while the sulfurylase gene (*moeB-1* CLJU_RS09840) was upregulated with both H_2_/CO_2_ and syngas (Supplementary Table S7). Another pair of sulfur carrier protein and sulfurylase genes nearby (*moaD-2* CLJU_RS09920, *moeB-2* CLJU_RS09925) were upregulated with H_2_/CO_2_ only (Supplementary Table S7). Other genes of molybdopterin biosynthesis were less upregulated (Supplementary Table S7). This suggests that when *C*. *ljungdahlii* grows with H_2_/CO_2_, incorporation of sulfur into molybdopterin may be increased to meet demand for molybdopterin-containing cofactors. Genes for molybdopterin-containing enzymes were also upregulated (Supplementary Discussion S2 and below).

### Indications of sulfur limitation under lithotrophic conditions

Several genes for uptake and metabolism of sulfur compounds were upregulated under lithotrophic conditions (Supplementary Table S8). One strongly upregulated operon (CLJU_RS13650-CLJU_RS13635) encodes components of a transporter with 32–51% identity to a putative *S*-methylcysteine transporter of *B*. *subtilis* ^33^ and a putative cystathionine beta-lyase with 37% protein sequence identity to PatB of *B*. *subtilis* ^35^. On the 5′ side of this operon is another upregulated operon (CLJU_RS13670-CLJU_RS13655); it encodes components of a transporter with 29–41% identity to the D-methionine and high-affinity L-methionine transporter of *E*. *coli* ^36^ and 30–37% identity to the D-methionine, L-methionine and methionine-sulfoxide transporter of *B*. *subtilis* ^37^, as well as a racemase/dehydratase. Upregulation of these genes (Supplementary Table S8) suggests that under lithotrophic conditions, *C*. *ljungdahlii* attempts to degrade thioethers of cysteine and/or homocysteine (e.g. methionine), possibly to access sulfur.

Three genes with 39–42% protein sequence identity to subunits of anaerobic sulfite reductase in *Salmonella enterica* Typhimurium^38^ (CLJU_RS11690*-*CLJU_RS11680) were upregulated with syngas but downregulated with H_2_/CO_2_ (Supplementary Table S8). These genes are strongly upregulated with NO_3_^−^ as the nitrogen source, and have been suggested to encode nitrite reductase^18^. On the 3′ side of these genes, four genes (CLJU_RS11675- CLJU_RS11660) encoding a candidate proton/nitrate symporter and components of a molybdopterin-dependent candidate assimilatory nitrate reductase^18^ were upregulated with syngas and downregulated with H_2_/CO_2_ (Supplementary Table S8). Thus, *C*. *ljungdahlii* responds differently to syngas and to H_2_/CO_2_ in terms of expression of genes for assimilation of sulfite and/or nitrate. These differences may prove important for industrial-scale applications of *C*. *ljungdahlii* with different electron donors and sources of sulfur and nitrogen.

Five genes in an operon (CLJU_RS12535- CLJU_RS12555) encoding a molybdopterin-dependent oxidoreductase of the dimethylsulfoxide reductase family, including a flavoprotein subunit with 41% protein sequence identity to that of anaerobic sulfite reductase in *Salmonella enterica* Typhimurium^38^, were upregulated with syngas and with H_2_/CO_2_ (Supplementary Table S8). The function of this putative complex, possibly in sulfoxide metabolism, is an interesting topic for future research.

Sulfur starvation can be simulated by the presence of chromate, which competes with sulfate uptake and assimilation (although *C*. *ljungdahlii* cannot assimilate sulfate^24^) and causes oxidative stress that depletes cysteine-based electron carriers^39^. Chromate efflux transporters consist of a pair (or fusion) of paralogous proteins with opposite topology^40,41^. In *C*. *ljungdahlii*, these two genes were upregulated with syngas and with H_2_/CO_2_ (Supplementary Table S8). Both have stronger BLAST hits to subunit 2, but topology prediction by the positive-inside rule^42^ indicates that CLJU_RS17795 encodes subunit 1 and CLJU_RS17790 encodes subunit 2. This upregulation of chromate efflux, together with the preceding observations, indicates that *C*. *ljungdahlii* is sulfur-limited under lithotrophic conditions. This may be an important consideration for biofuel production, which might be maximized by control of the levels of enzymes in *C*. *ljungdahlii* by the availability of sulfur. Notably, sulfur uptake and metabolism proteins that are more abundant in solventogenesis *versus* acidogenesis from syngas^24^ are different from these genes upregulated in lithotrophic *versus* organotrophic growth.

### Upregulation of quorum sensing and sporulation genes under lithotrophic conditions

**Figure 4:**
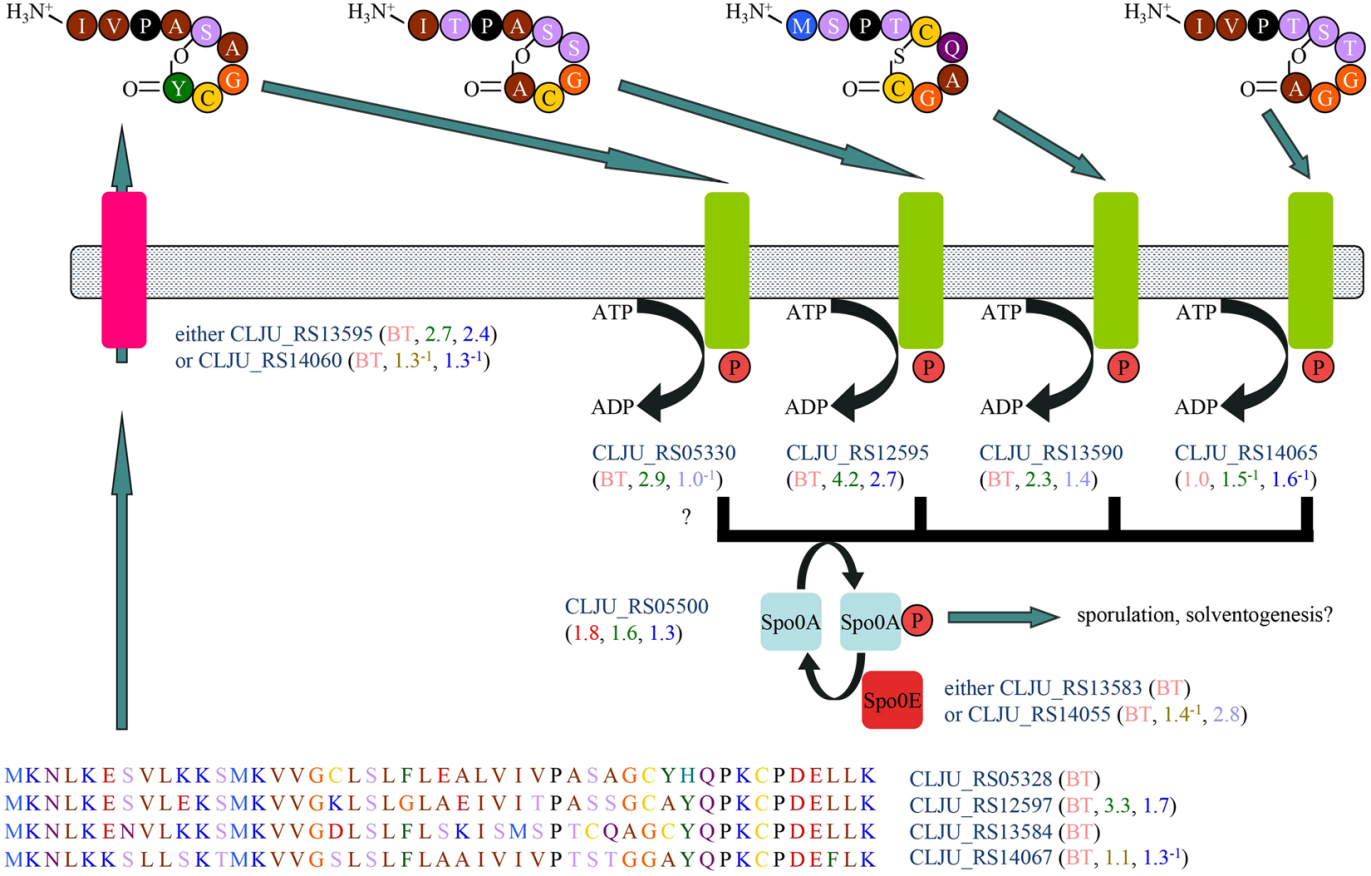
Upregulation of quorum sensing genes under lithotrophic conditions. Four autoinducer precursor proteins may be processed into peptide lactones by membrane proteins during secretion. (Lactone structures illustrated are predictions based on LamD of *L*. *plantarum*; actual structures are unknown). The secreted autoinducer lactones are predicted to be sensed by histidine kinases, which activate Spo0A. Spo0E dephosphorylates Spo0A. Accumulation of phosphorylated Spo0A commits the cell to behaviours such as sporulation, solventogenesis, or biofilm formation.

Biofilm formation by *C*. *ljungdahlii* is a factor that could improve the efficacy of commodity chemical production from syngas or by electrosynthesis^20^. The genome of *C*. *ljungdahlii* encodes four sensor histidine kinases (Fig. 4) that are homologs of AgrC of *Clostridium acetobutylicum* (20–22% protein sequence identity), the sensor of a peptide thiolactone autoinducer-dependent signaling system controlling sporulation and synthesis of the storage polymer granulose^43^, and LamC of *Lactobacillus plantarum* (18–21% protein sequence identity), the putative sensor for peptide thiolactone autoinducer-dependent control of biofilm formation^44^. These sensor kinases are 72–80% identical to each other in protein sequence and are each encoded next to genes for precursor proteins of peptide (thoi)lactone autoinducers (two of which were discovered through this work). Membrane proteins for autoinducer processing are also encoded near two of the kinases. Several of these quorum sensing genes were upregulated with H_2_/CO_2_ and with syngas (Fig. 4). Notably, no response regulators are encoded next to any of the four sensor histidine kinase genes, but genes next to two of them encode homologs of Spo0E, a phosphatase that deactivates the Spo0A protein that regulates both sporulation and solventogenesis in *Clostridium beijerinckii* and *Clostridium cellulolyticum* ^45^. These orphan kinases are candidates to activate and deactivate Spo0A, as in *C*. *acetobutylicum* ^46^. Numerous genes that were upregulated under lithotrophic conditions have been implicated in sporulation or cell wall biogenesis (Supplementary Table S9), indicating that the change in growth mode causes global changes in physiology. Investigation of the quorum sensing signaling systems for possible control of sporulation, biofilm formation, and solventogenesis by *C*. *ljungdahlii* may lead to improved designs for large-scale production of biofuels and commodity chemicals.

In *B*. *subtilis*, glutamine-hydrolyzing asparagine synthetase is expressed specifically during sporulation, for which it is required^47^. In *C*. *ljungdahlii*, the homologous gene (*asnO* CLJU_RS13170) was upregulated with H_2_/CO_2_ (Supplementary Table S9), but so was the gene for ammonia-dependent asparagine synthetase (CLJU_RS03315). Asparaginyl-tRNA (made by transamidation, however) has been proposed to be a signal in the switch from acidogenesis to solventogenesis in *C*. *acetobutylicum* ^48^. Future experiments may determine whether asparagine or asparaginyl-tRNA is also a signal in *C*. *ljungdahlii*.

### Upregulation of various membrane-associated proteins

Genes for transfer of methyl groups from unidentified substrates into the Wood-Ljungdahl pathway were upregulated under lithotrophic conditions (Supplementary Discussion S3, Supplementary Table S10). Several strongly upregulated genes encode membrane proteins (Supplementary Table S11), although their exact functions cannot be predicted yet. A membrane protein of the major facilitator superfamily, possibly a transporter, is encoded by CLJU_RS14525, which was the third most upregulated gene with H_2_/CO_2_ according to RNA-Seq and the first most upregulated according to the microarray, and the sixteenth most upregulated with syngas (Supplementary Table S11). Ten adjacent genes were also strongly upregulated (Supplementary Table S11), including a poly-alpha-D-galacturonosidase gene (*pehX* CLJU_RS14480) with 37% protein sequence identity to that of *Erwinia chrysanthemi* ^49^. Poly-alpha-D-galacturonate is a component of plant cell walls; the upregulation of a gene for its degradation suggests that in the presence of H_2_/CO_2_ or syngas, *C*. *ljungdahlii* may prepare to encounter plant cell matter. Considering that *C*. *ljungdahlii* was isolated from chicken yard waste^1^, it is possible that the digestive tract of a chicken is a natural environment where *C*. *ljungdahlii* degrades plant cell matter while growing lithotrophically with H_2_, CO_2_, and CO.

### Downregulation of fructose-specific and glycolytic enzyme genes

**Figure 5:**
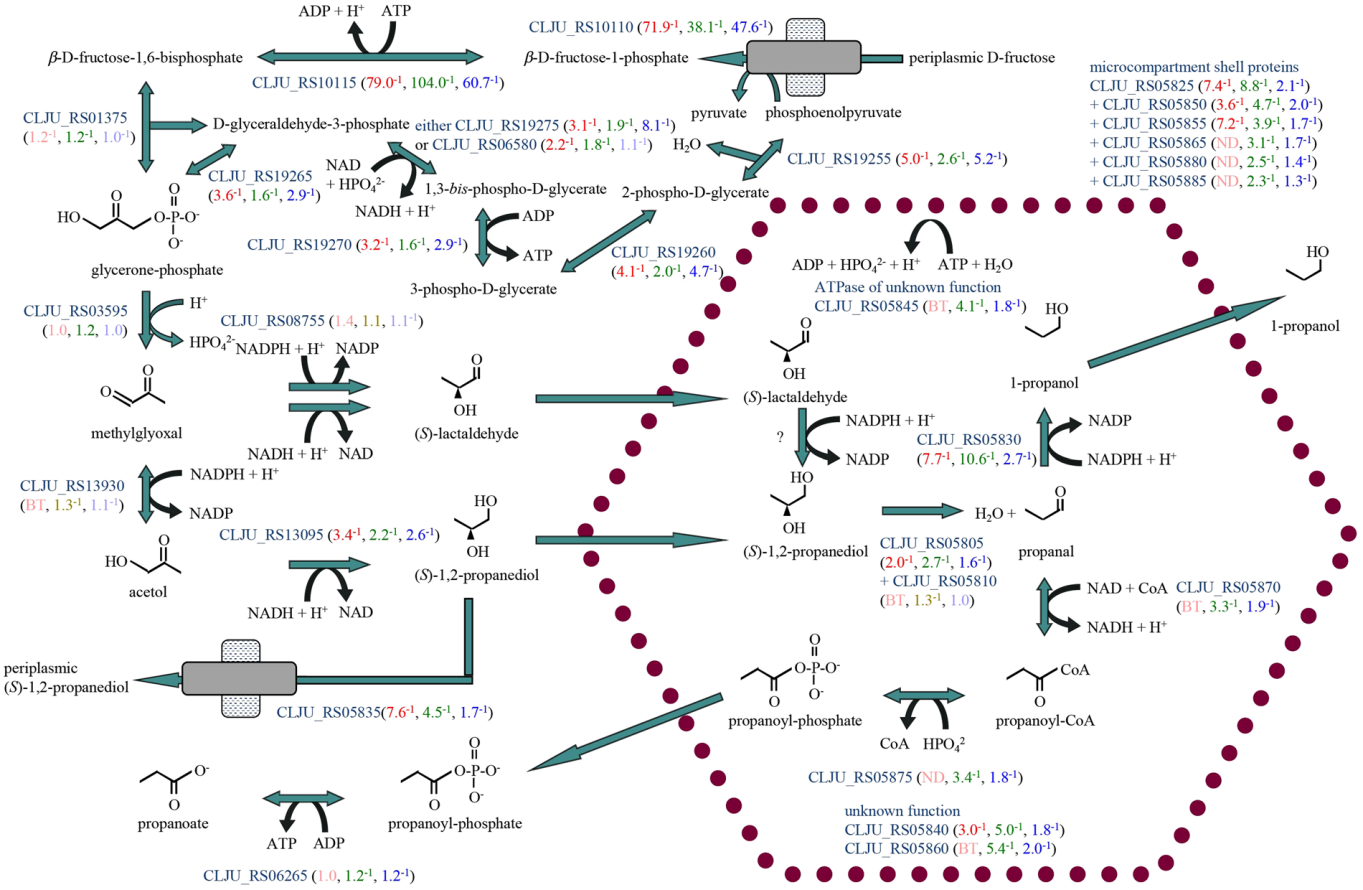
Downregulation of enzymes of glycolysis and methylglyoxal detoxification under lithotrophic conditions. Uptake of fructose is driven by conversion of phosphoenolpyruvate into pyruvate at the end of the glycolytic pathway, which otherwise yields one more ATP. Methylglyoxal synthase functions to release phosphate from glycerone-phosphate, producing methylglyoxal for reduction to *(S)*-1,2-propanediol, which is disproportionated to propanoyl-phosphate and 1-propanol inside a microcompartment within the cytoplasm.

Predictably, the three most downregulated genes under lithotrophic conditions compared to organotrophic growth on fructose encode the fructose transport phosphotransferase system-specific membrane protein (*fruA* CLJU_RS10110), the enzyme fructose-1-phosphate 6-kinase (*fruK* CLJU_RS10115) that is only required for utilization of exogenous fructose (Fig. 5), and the fructose-1-phosphate-responsive transcriptional regulator (*fruR* CLJU_RS10120, Supplementary Table S2). Genes for the glycolytic enzymes were also downregulated (Fig. 5; as with CO/CO_2_^17^ and H_2_/CO_2_^18^).

### Downregulation of glycerol/(*S*)–1,2-propanediol metabolism genes

One strongly downregulated gene encodes an NADPH-dependent aldehyde reductase (*pdgQ* CLJU_RS05830) with 40% protein sequence identity to the acetaldehyde/butanal reductase of *Clostridium saccharobutylicum* ^50^. The location of this gene among genes for shell proteins of a microcompartment and for glycerol/(*S*)–1,2-propanediol dehydratase, most of which were downregulated with syngas and with H_2_/CO_2_ (Fig. 5), suggests that it functions as 3-hydroxypropanal/propanal reductase. These genes may be expressed during growth on fructose to dispose of methylglyoxal, a toxic byproduct of glycolysis. Methylglyoxal may be reduced to (*S*)–1,2-propanediol in the cytoplasm and then disproportionated to 1-propanol and propanoyl-phosphate within the microcompartment (Fig. 5). Efforts to produce solvents such as 1-propanol, 1-butanol, or 1,3-propanediol with *C*. *ljungdahlii* grown lithotrophically may benefit from genetic engineering to abolish downregulation of these genes.

### Downregulation of pyrimidine and purine biosynthesis, uptake and salvage genes

**Figure 6:**
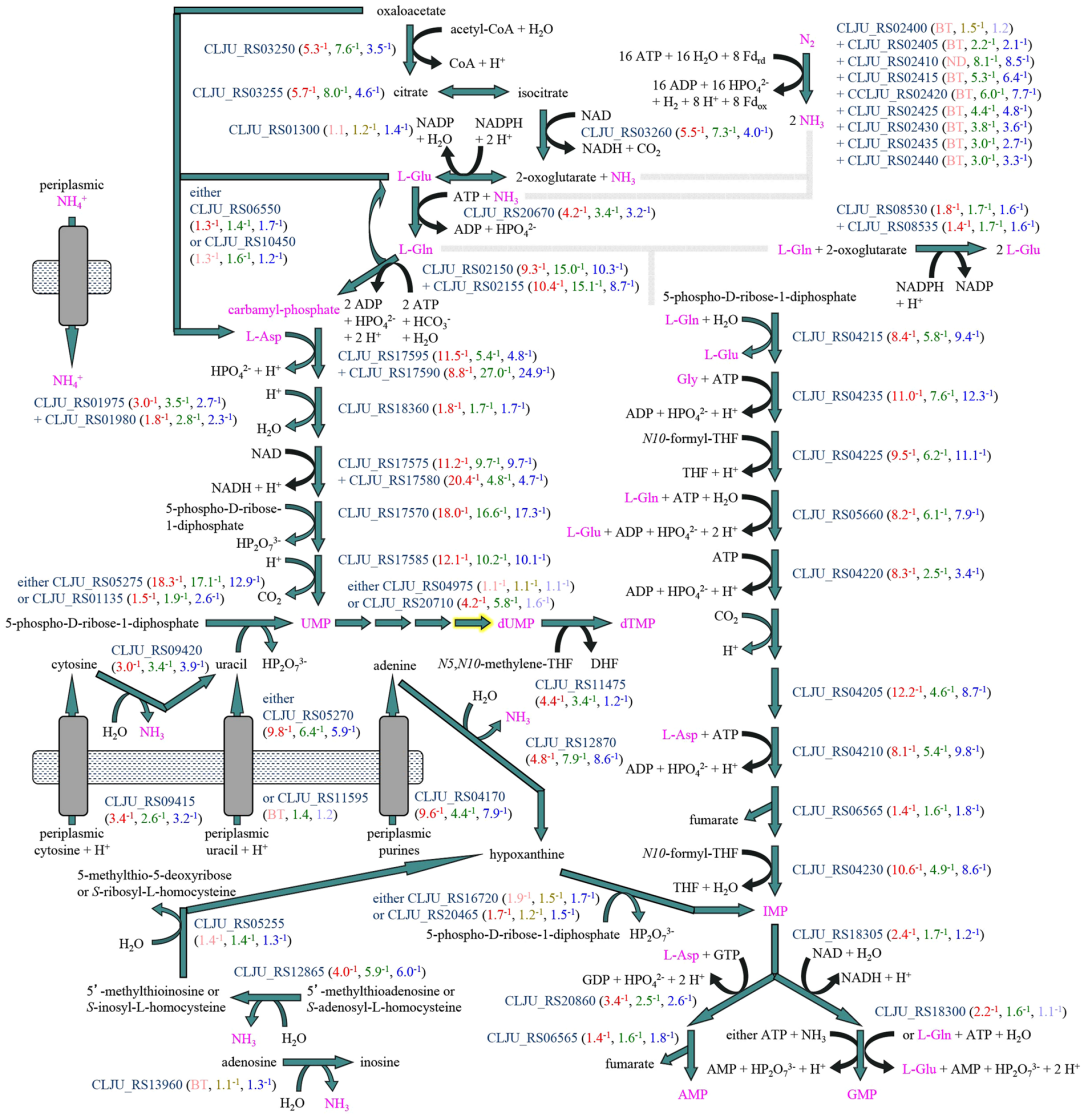
Downregulation of pyrimidine and purine biosynthesis, salvage and uptake under lithotrophic conditions. Nitrogen being assimilated is shown in pink. Biosynthesis of pyrimidines and purines depends upon ammonium uptake, nitrogen fixation to ammonia and synthesis of oxaloacetate and 2-oxoglutarate, which are the carbon backbones of aspartate and glutamate/glutamine. Pyrimidine biosynthesis begins with one of two isozymes of carbamyl-phosphate synthase, different from the one in the urea cycle (Fig. 2).

Two operons encoding enzymes of pyrimidine biosynthesis and purine biosynthesis were among the most downregulated genes under lithotrophic conditions (Fig. 6). This is consistent with the expectation that growth in the absence of an organic carbon source, being slower, requires less nucleic acid synthesis for genome replication and gene transcription. Downregulation with syngas and with H_2_/CO_2_ was also observed for genes of pyrimidine and purine biosynthesis, salvage and uptake in other locations in the genome (Fig. 6), including one of two isozymes of carbamyl-phosphate synthase. This downregulated isozyme appears to be dedicated to pyrimidine biosynthesis, while the other isozyme that was upregulated (Fig. 2) is dedicated to the urea cycle. Genes for biosynthesis of glutamate, glutamine and aspartate, which are substrates for pyrimidine and purine biosynthesis, were also downregulated, along with genes for ammonium uptake and nitrogen fixation (Fig. 6). Altogether, these observations indicate a contrast between assimilation of nitrogen into nucleotides under organotrophic conditions and disposal of nitrogen as ammonia and urea under lithotrophic conditions. Genes for phosphate uptake and dephosphorylation of nucleotides were differentially regulated (Supplementary Discussion S4, Supplementary Table S12).

A homolog of the phosphoribosylaminoimidazolecarboxamide formyltransferase enzyme of purine biosynthesis is encoded by a gene (CLJU_RS10900) that was the fourth most downregulated gene (65.3-fold) with H_2_/CO_2_ according to RNA-Seq and the fifth most downregulated (26.2-fold) according to the microarray, consistent with a previous report^18^, but only 1.6-fold downregulated with syngas. The protein sequence divergence of this gene and its location apart from known purine biosynthesis genes suggest that it belongs to another pathway. On its 5′ side is a *pfl* RNA motif, hypothesized to be a riboswitch to regulate one-carbon unit metabolism^51^, and on its 3′ side is an adenosylcobamide-responsive riboswitch (transcribed with CO/CO_2_)^17^ in an antiparallel orientation, suggesting that antisense transcription through CLJU_RS10900 may occur when vitamin B12 is limiting. It will be interesting to investigate this gene product, which was abundant in a syngas proteomic sample^24^, for a role in one-carbon unit metabolism.

Genes for biosynthesis of threonine, a precursor of vitamin B12, were downregulated and high-threonine-content protein genes that were downregulated included some that are adjacent to adenosylcobamide-responsive riboswitches (Supplementary Discussion S5, Supplementary Tables S13, S14 and S15).

### Downregulation of cytoplasmic redox-active protein genes

Several downregulated genes are predicted to encode cytoplasmic redox-active proteins (Supplementary Table S3), such as a rubredoxin domain oxidoreductase (*hrb* CLJU_RS09545; also downregulated with CO/CO_2_^17^) with 47% protein sequence identity to Hrb of *Moorella thermoacetica* ^52^. The Hrb protein oxidizes NADH and reduces rubredoxin:oxygen/nitric oxide oxidoreductase (*roo* CLJU_RS10770), for which the gene was also downregulated. Both genes were previously reported to be upregulated with H_2_/CO_2_ *versus* fructose^18^ and under O_2_ stress^19^. Downregulation of two genes that encode homologs of the iron-sulfur-oxygen hybrid cluster protein that is thought to detoxify an unidentified reactive compound in response to nitrous oxide stress^53^ was more obvious with H_2_/CO_2_ than with syngas.

### Downregulation of membrane-associated redox-active proteins with syngas

Other downregulated genes encode membrane-associated redox-active proteins (Supplementary Table S4). Two genes that were downregulated with syngas (CLJU_RS14585, CLJU_RS14610) are notable for their protein sequence similarity to NapH, the ubiquinol-oxidizing subunit of the periplasmic nitrate reductase of *E*. *coli* ^25^, and the predicted locations of iron-sulfur cluster-binding motifs within transmembrane segments or outside the cytoplasmic membrane. It would be interesting to study the roles of these genes in membrane-associated redox reactions, possibly including how electrical current is consumed.

## Conclusions

This study compared the transcriptomic profiles of *C*. *ljungdahlii* growing lithotrophically with H_2_/CO_2_ or with syngas and organotrophically with fructose. The genes that were observed to be differentially regulated offer insights into physiological changes such as quorum sensing and sporulation, the roles of specific metabolic pathways such as the urea cycle, and differential usage of amino acids such as histidine. Numerous redox-active proteins of the cytoplasm and membrane were identified that may be investigated for roles in electron transfer across the cell wall of *C*. *ljungdahlii*.

## Materials and Methods

### Growth of *C. ljungdahlii*

*C*. *ljungdahlii* DSM 13528 (ATCC 55383) was purchased from the German Collection of Microorganisms and Cell Cultures (DSMZ). For general propagation, *C*. *ljungdahlii* cells were grown anaerobically at 37 °C in PETC 1754 medium supplemented with 1 mM L-cysteine (pH 7) and fructose at 5 g/L. For transcriptomic studies, *C*. *ljungdahlii* cells were grown in DSMZ 879 medium supplemented with 0.04% L-cysteine, 0.04% sodium sulfide and 0.1% sodium bicarbonate, inoculated to OD600~0.05 with precultures grown to OD600~0.3–0.5 in DSMZ 879 medium under the same conditions (with fructose, H_2_/CO_2_, or syngas). For organotrophic growth, fructose at 5 g/L was added to DSMZ 879 medium. For lithotrophic growth, pressure tubes containing 5 ml instead of 10 ml of DSMZ 879 medium were used. For lithotrophic growth on H_2_/CO_2_, the headspace of culture tubes was replaced with H_2_/CO_2_ (80/20) and pressurized to 20 psi after inoculation. For growth on syngas (H_2_:CO_2_:CO ratio 45:5:50), the headspace was replaced with syngas and pressurized to 20 psi. Cultures that grew lithotrophically were laid flat while shaking at 100 rpm during incubation at 37 °C. In experiments to determine the OD600 range of mid-log growth, the headspace was pressurized with the appropriate gas mix every 24 hours after cell turbidity reached OD600~0.3.

### RNA isolation

Total RNA was isolated from mid-log DSMZ 879-grown cultures of OD600~0.4–0.5 with fructose, OD600~0.2 with H_2_/CO_2_, and OD600~0.2 with syngas. When cultures reached the appropriate cell density, two volumes of RNAprotect Bacteria Reagent (Qiagen, USA) were anaerobically added to the cultures, mixed by vortex for 30 seconds, and incubated at room temperature for 10 minutes before collecting cells at 10,000 × *g* for 5 minutes. Cell pellets were snap-frozen in liquid nitrogen immediately after centrifugation and kept at −80 °C until RNA extraction. Total RNA was isolated with the RiboPure bacteria kit (Ambion, USA) following the manufacturer’s instructions. The integrity and quantity of isolated total RNA were checked using a Bio-Rad Experion system (Bio-Rad, USA).

### cDNA synthesis

cDNA was synthesized from total RNA with the TransPlex Complete Whole Transcriptome Amplification kit (Sigma-Adrich, USA) following the manufacturer’s instructions.

### Microarray analysis and chip design

Triplicate total RNA samples from each growth condition were collected for DNA microarray analysis. The microarray was designed using the published genome sequence (NC_014328.1) and included 4184 CDS from the *C*. *ljungdahlii* ATCC55383 chromosome. In addition, probes for genes for the butanol biosynthesis pathway from *C*. *acetobutylicum* and other bacteria were also included in the microarray design (GeneID:1119056, 1118891, 1118895, 1118894, 1118892, 1118893, 1116040, 1116167, 1119481, 1119480, 1119259, 1119258, 2741560, 946727, 4413431, 5294993, 5291557, 124221917, 5292938, and 9265716). The probe sets were designed by NimbleGen (USA) according to the company’s protocols and algorithms, and all probes were manufactured directly onto the slides via photolithographic synthesis of 45-to-60-mer oligonucleotides. For every gene sequence, up to 8 individual probes were designed to cover the gene sequence of interest (for detailed information refer to Supplementary Table S16). Each probe was synthesized in two replicates located in different array regions (BLOCK1 and BLOCK2), resulting in 66,800 unique probe sets covering 4204 genes. Duplicated genes in the *C*. *ljungdahlii* genome that were omitted from probe sets are listed in Supplementary Table S16.

### mRNA enrichment and Illumina library preparation for RNA-Seq

Total RNA isolated from triplicate cultures (H_2_/CO_2_ and fructose, different from the cultures used for microarray analysis) was used for an enrichment of mRNA using the *MICROBExpress* kit (Ambion, USA), following the manufacturer’s protocol. Quality and quantity of the enriched mRNA were checked by analyzing aliquots of mRNA using the Experion RNA HiSens kit (Bio-Rad, USA), following the manufacturer’s protocol (Supplementary Fig. S1). The enriched mRNA samples thus obtained were used to construct Illumina libraries (3 × 2 samples) using the ScriptSeq™ v2 RNA-Seq Library Preparation Kit (Epicentre, USA), which enables directional sequencing, following the manufacturer’s protocol. Briefly, 200 ng of the total mRNA were chemically fragmented and converted into single–stranded cDNA with random hexamer priming. Next, the single-stranded cDNA was 3′-terminal-tagged. Di-tagged cDNA products were then amplified and enriched by using individual adapters containing unique hexameric indices/barcodes with a final 10-cycle PCR reaction. Altogether, six enriched and purified libraries with unique barcodes (representing three replicates with either H_2_/CO_2_ or fructose) were obtained, mixed in an equimolar concentration, and used for hybridization in a HiSeq 2000 flow cell for single-end sequencing.

### Assembly of Illumina reads

All of the raw data generated by Illumina sequencing were quality checked by visualization of base quality scores and nucleotide distributions. Then the sequences were sorted out by trimming of reads and read filtering based on base quality score and sequence properties such as primer contaminations, N content and GC bias using PRINSEQ^54^. All good quality mRNA sequence reads were assembled and mapped against the published genome of *C*. *ljungdahlii*, DSM 13528 (NC_014328) using ARRAY STAR (DNA star, USA). Reads belonging to 16 S/23 S rRNA, reads that matched more than one segment of the genome and reads with more than two mismatches were discarded. The remaining mRNA reads were reanalyzed and normalized with the RPKM (reads assigned per kilobase of target per million mapped reads) method^55,56^ using ARRAY STAR. Reads from biological replicates were first compared with each other graphically after mapping onto the template genome. Biological replicates were highly reproducible (Supplementary Fig. S1). Therefore, reads from biological replicates were merged and averaged for each experimental condition. Expression levels were considered significant only when the log_2_ RPKM value was ≥5 (median log_2_ RPKM value) (Supplementary Fig. S2; Supplementary Table S1) in one of the experiments and were used to calculate fold change (Supplementary Fig. S2). The significance level for downregulation or upregulation was calculated at *p* = 0.05.

### Data Availability

Microarray data and RNA-Seq sequence reads have been submitted to the EMBL databases under accession nos. E-MTAB-3806 and PRJEB9771, respectively.

## Electronic supplementary material

Supplementary information

Supplementary Tables (S1- S16)
