# Supplementary material for: Transcriptomic profiles of Clostridium ljungdahlii during lithotrophic growth with syngas or H2 and CO2 compared to organotrophic growth with fructose
Source: Sci Rep. 2017 Oct 13;7:13135. doi: 10.1038/s41598-017-12712-w (PMC5640608; doi:10.1038/s41598-017-12712-w)
Supplement: Supplementary file 1 — Supplementary information [file 41598_2017_12712_MOESM1_ESM.pdf]

Supplementary Information

Transcriptomic profiles of *Clostridium ljungdahlii* during lithotrophic growth with syngas or H_2 and CO_2 compared to organotrophic growth with fructose

Running title: *C. ljungdahlii* transcriptomics

Muktak Aklujkar[1, 2]†* (maklujkar@unr.edu)

Ching Leang[1, 3]† (Ching.Leang@lanzatech.com)

Pravin M. Shrestha[1, 4]*† (pravin@berkeley.edu)

Minita Shrestha[1, 5] (minita@lbl.gov)

Derek R. Lovley[1] (dlovley@microbio.umass.edu)

[1]University of Massachusetts Amherst, Amherst, MA 01003

[2]University of Nevada, Reno, NV 89557-0352

[3]LanzaTech, Skokie, IL 60077

[4]Energy Biosciences Institute, University of California, Berkeley, CA 94720

[5]Earth and Environmental Sciences, LBNL, Berkeley, CA 94720

†All three authors contributed equally

[*]Corresponding author

Phone: (510) 666-2766

Fax: (510) 642-4995

Supplementary discussions

Supplementary Discussion S1. Salvaging and detoxification of urea cycle intermediates.

Supplementary Discussion S2. Upregulation of molybdopterin-containing enzymes under lithotrophic conditions.

Supplementary Discussion S3. Mobilization of methyl groups.

Supplementary Discussion S4. Phosphate uptake and nucleotide dephosphorylation.

Supplementary Discussion S5. Downregulation of threonine biosynthesis genes, high-threonine-content genes, and cell surface proteins.

Supplementary Tables (See excel table)

Supplementary Table S1. Differentially regulated genes, ranked by fold change. A log2 value of "undef" indicates that a gene transcript was not detected in RNA from a particular growth condition.

Supplementary Table S2. Differentially regulated transcriptional regulator and signalling system genes. Fold changes are given; those for which the *p* values were not significant are in grey. Only genes with a change twofold or greater in at least one experiment are shown.

Supplementary Table S3. Upregulation and downregulation of predicted cytoplasmic redox-active protein genes during lithotrophic growth. Fold changes are given; those for which the *p* values were not significant are in grey.

Supplementary Table S4. Upregulation and downregulation of membrane-associated oxidoreductase gene clusters under lithotrophic conditions. Fold changes are given; those for which the *p* values were not significant are in grey.

Supplementary Table S5. Differentially regulated genes of peptide and amino acid uptake and degradation. Fold changes are given; those for which the *p* values were not significant are in grey.

Supplementary Table S6. High-histidine-content genes that are upregulated under lithotrophic conditions. Fold changes are given; those for which the *p* values were not significant are in grey. Only genes with at least one change twofold or greater are shown.

Supplementary Table S7. Upregulation of genes for molybdopterin-Mo/W cofactor biosynthesis under lithotrophic conditions. Fold changes are given; those for which the *p* values were not significant are in grey.

Supplementary Table S8. Upregulation of sulfur metabolism genes under lithotrophic conditions. Fold changes are given; those for which the *p* values were not significant are in grey.

Supplementary Table S9. Upregulation and downregulation of sporulation and cell wall biogenesis genes under lithotrophic conditions. Fold changes are given; those for which the *p* values were not significant are in grey. Only genes with at least one change twofold or greater are shown.

Supplementary Table S10. Upregulation of corrinoid-dependent methyl group transfer genes and folate uptake genes and downregulation of folate biosynthesis genes under lithotrophic conditions. Fold changes are given; those for which the *p* values were not significant are in grey.

Supplementary Table S11. Strong upregulation and strong downregulation of membrane-associated proteins of unknown function under lithotrophic conditions. Fold changes are given; those for which the *p* values were not significant are in grey.

Supplementary Table S12. Downregulation of phosphate uptake transporter gene clusters and upregulation of a short-chain exopolyphosphatase under lithotrophic conditions. Fold changes are given; those for which the *p* values were not significant are in grey.

Supplementary Table S13. Downregulation of gene clusters for threonine biosynthesis and activation under lithotrophic conditions. Fold changes are given; those for which the *p* values were not significant are in grey.

Supplementary Table S14. High-threonine-content genes that are downregulated under lithotrophic conditions. Fold changes are given; those for which the *p* values were not significant are in grey. Only genes with at least one change twofold or greater are shown.

Supplementary Table S15. Downregulation of a gene cluster encoding cell surface proteins under lithotrophic conditions. Locations of the ten adenosylcobamide-responsive riboswitches

that may regulate the expression level of these genes are indicated. Fold changes are given; those for which the *p* values were not significant are in grey.

Supplementary Table S16. Probes for microarray.

Supplementary Figures

Supplementary Figure S1. (a) Highly reproducible mRNA extraction as shown by electropherogram output of Experion RNA HiSen kit. As a representative example, mRNA extracts from one of the samples of *C. ljungdahlii* grown on H_2/CO_2 are shown in the figure. (b-c) Scatter plots showing comparison of gene expression levels in two representative replicate samples of *C. ljungdahlii* grown on H_2/CO_2 (b) and fructose (c).

Supplementary Figure S2. (a) Median $\log_2$ RPKM value calculated for *C. ljungdahlii* grown on H_2/CO_2. Only the gene expression levels ≥ median $\log_2$ RPKM values were treated as the significant expression. (b) Scatter plot showing comparison of gene expression levels of *C. ljungdahlii* grown on H_2/CO_2 *versus* fructose. Each data point on the scatter plot represents an individual gene and is plotted based on its expression level in both of the selected experiments. (c) Heat map comparison of global gene expression levels of *C. ljungdahlii* grown on H_2/CO_2 and fructose.

Supplementary discussion S1.

Salvaging and detoxification of urea cycle intermediates. As an alternative to arginase, *C. ljungdahlii* possesses a set of genes for ammonia-producing, ATP-yielding fermentation of arginine to ornithine (Figure 2), and a partial second set - lacking arginine deiminase - for fermentation of citrulline to ornithine (Figure 2). Of the first set, the genes for arginine deiminase (*arcA* CLJU_RS04570), ornithine carbamyltransferase (*arcB-1* CLJU_RS04575), the arginine/ornithine antiporter (*arcD-1* CLJU_RS04580) with 48% protein sequence identity to that of *Pseudomonas aeruginosa*[1], and carbamate kinase (*arcC-1* CLJU_RS04585) were downregulated with H_2/CO_2 (Figure 2). Of the second set, the putative citrulline/ornithine antiporter gene (*arcD-2* CLJU_RS13825) with 46% protein sequence identity to the arginine/ornithine antiporter of *P. aeruginosa*[1] was not upregulated, but the ornithine carbamyltransferase (*arcB-2* CLJU_RS13835) and carbamate kinase (*arcC-2* CLJU_RS13830) genes were upregulated with syngas and with H_2/CO_2 (Figure 2; as previously reported with H_2/CO_2[2]). These enzymes may be important if the urea cycle stalls for lack of aspartate or if too much ammonia has been sequestered, because they can revert citrulline to ornithine plus carbamyl-phosphate, recover one-third of the expended ATP, and release ammonia (Figure 2). The gene for cyanate lyase (CLJU_RS07235) was upregulated with syngas and with H_2/CO_2 (Figure 2), indicating that carbamyl-phosphate may accumulate under lithotrophic conditions and undergo nonenzymatic base-catalyzed elimination of phosphate, producing cyanate that must be detoxified by cyanate lyase.

1. Verhoogt, H. J. *et al. arcD*, the first gene of the *arc* operon for anaerobic arginine catabolism in *Pseudomonas aeruginosa*, encodes an arginine-ornithine exchanger. *J*

Bacteriol **174,** 1568–1573 (1992).

2. Nagarajan, H. *et al.* Characterizing acetogenic metabolism using a genome-scale metabolic reconstruction of *Clostridium ljungdahlii*. *Microb. Cell Fact.* **12,** 118 (2013).

Supplementary discussion S2.

Upregulation of molybdopterin-containing enzymes under lithotrophic conditions. Among the molybdopterin biosynthesis genes are encoded two aldehyde:ferredoxin oxidoreductases (*aor-1* CLJU_RS09865 and *aor-2* CLJU_RS09915, sharing 79% protein sequence identity), which are molybdopterin cofactor-containing enzymes. Both genes were upregulated with H_2/CO_2, and the *aor-1* gene was also upregulated with syngas (Figure 1). With H_2 as the electron donor and without the hypothetical coupled reduction of methylene-THF and ferredoxin with NADH[1], the Wood-Ljungdahl pathway ending with acetyl-CoA, acetyl-phosphate, and acetate has a predicted net energy yield of 0.41 ATP, while the pathway ending with reduction of acetyl-CoA with NADH to acetaldehyde and reoxidation to acetate by aldehyde:ferredoxin oxidoreductase has a predicted net energy loss of 0.05 ATP. *C. ljungdahlii* may sacrifice a fraction of the flux through the Wood-Ljungdahl pathway in this manner to maintain a pool of reduced ferredoxin sufficient to favour the reaction catalyzed by carbon monoxide dehydrogenase. However, this fraction is likely to be small because the enzymes that could reduce acetyl-CoA with NADH were generally downregulated (Figure 1).

In addition to formate dehydrogenase, *C. ljungdahlii* possesses a formate hydrogen-lyase, the Hfn or Hyt complex (Figure 1)[2], by which electrons are predicted to be transferred from a [FeFe]-hydrogenase catalytic subunit (*hfnI* CLJU_RS03480) through a series of five iron-sulfur cluster-binding subunits either to NADP and Fd_{ox} or to a formate dehydrogenase catalytic

subunit containing selenocysteine and a molybdopterin cofactor (*hfnU* CLJU_RS03435). Remarkably, formate hydrogen-lyase gene expression levels were similar under lithotrophic conditions whether the electron donor was H_2 alone or a mixture of H_2 and CO in syngas (Figure 1). A protein with 70% sequence identity to HfnU, encoded separately (*hfnC* CLJU_RS04405), appears to be an alternative formate dehydrogenase catalytic subunit for the complex, with cysteine rather than selenocysteine in the molybdopterin-containing active site. The *hfnC* gene was upregulated more than the other *hfn* genes with syngas and with H_2, suggesting that flux through formate hydrogen-lyase might be limited by the ability of *C. ljungdahlii* to produce the selenoprotein HfnU.

1. Köpke, M. *et al. Clostridium ljungdahlii* represents a microbial production platform based on syngas. *Proc. Natl. Acad. Sci. U. S. A.* **107,** 13087–13092 (2010).
2. Wang, S. *et al.* NADP-specific electron-bifurcating [FeFe]-hydrogenase in a functional complex with formate dehydrogenase in *Clostridium autoethanogenum* grown on CO. *J. Bacteriol.* **195,** 4373–4386 (2013).

Supplementary discussion S3.

Mobilization of methyl groups. Enzymes that transfer methyl groups from various substrates to the cobalt centres of corrinoid-binding proteins and enzymes that transfer cobalt-centre-bound methyl groups to THF offer an advantage to acetogens such as *C. ljungdahlii* because they provide ATP through the Wood-Ljungdahl pathway and because the substrate may become fermentable upon demethylation. Remarkably, nineteen genes of *C. ljungdahlii* encode proteins similar to the cob(I)amide methyltransferase domain of chloromethane--cob(I)amide methyltransferase of *Methylobacterium chloromethanicum*[1]. Several of these genes were upregulated under lithotrophic conditions (Supplementary Table S10). It may be valuable to

discover the identities of their substrates. These methyltransferases may interact with eight different methyl-Co(III)-5-hydroxybenzimidazolylcobamide-binding proteins, some of which were upregulated (Supplementary Table S10). Other upregulated genes linked to the methyltransferases encode transcriptional regulators of the PucR family, membrane proteins of the major facilitator superfamily that may be transporters, and a putative GTPase for nickel insertion into an enzyme (Supplementary Table S10). A candidate folate transporter gene[2] with a folate-responsive riboswitch on its 5' side[3] was upregulated, as was the dihydrofolate reductase gene (Supplementary Table S10), whereas genes for folate biosynthesis were either downregulated or not differentially regulated. Altogether, this pattern indicates that under lithotrophic conditions, *C. ljungdahlii* seeks to import folate for the Wood-Ljungdahl pathway and to mobilize methyl groups from diverse substrates. However, expression of genes encoding folate-dependent enzymes of the Wood-Ljungdahl pathway was not increased in lithotrophic modes of growth (Supplementary Table S1). This is consistent with earlier reports that these genes were not differentially regulated with H_2/CO_2[4] and upregulated 1.8-fold or less with CO/CO_2[5].

1. Studer, A., Stupperich, E., Vuilleumier, S. & Leisinger, T. Chloromethane: tetrahydrofolate methyl transfer by two proteins from *Methylobacterium chloromethanicum* strain CM4. *Eur J Biochem* **268,** 2931–2938 (2001).
2. Rodionov, D. A. *et al.* A novel class of modular transporters for vitamins in prokaryotes. *J Bacteriol* **191,** 42–51 (2009).
3. Ames, T. D., Rodionov, D. A., Weinberg, Z. & Breaker, R. R. A eubacterial riboswitch class that senses the coenzyme tetrahydrofolate. *Chem Biol* **17,** 681–685 (2010).
4. Nagarajan, H. *et al.* Characterizing acetogenic metabolism using a genome-scale metabolic reconstruction of *Clostridium ljungdahlii*. *Microb. Cell Fact.* **12,** 118 (2013).
5. Tan, Y., Liu, J., Chen, X., Zheng, H. & Li, F. RNA-seq-based comparative transcriptome analysis of the syngas-utilizing bacterium *Clostridium ljungdahlii* DSM 13528 grown autotrophically and heterotrophically. *Mol. Biosyst.* **9,** 2775–84 (2013).

Supplementary discussion S4.

Phosphate uptake and nucleotide dephosphorylation. Consistent with diminished nucleic acid synthesis under lithotrophic conditions, genes for phosphate uptake transporters were downregulated (Supplementary Table S12). In contrast, the *surE* gene (CLJU_RS17130), encoding a nucleoside-3'/5'-monophosphate phosphatase and short-chain exopolyphosphatase, was upregulated, suggesting that *C. ljungdahlii* under lithotrophic conditions may recycle intracellular phosphate instead of taking up more from its surroundings.

Surprisingly, of the two genes encoding isozymes of deoxyuridine-5'-triphosphate pyrophosphohydrolase, an enzyme required for conversion of uracil into thymine, transcripts were more abundant and microarray signals were more intense for CLJU_RS04975, which was not differentially regulated; the other gene CLJU_RS20710 was only downregulated with H_2/CO_2, not with syngas (Figure 6). An understanding of these two isozymes could be conducive to control of biomass production by limiting DNA replication in *C. ljungdahlii* to maximize the output of organic end products.

Supplementary discussion S5.

Downregulation of threonine biosynthesis genes, high-threonine-content genes, and cell surface proteins. Two genes for biosynthesis of threonine were strongly downregulated (Supplementary Table S13, as previously observed with H_2/CO_2 *versus* fructose[1]), suggesting that this amino acid may be especially important for organotrophic growth. The presence of a T-box antiterminator riboswitch on the 5' side of these genes suggests that their expression during growth on fructose may increase in response to deaminoacylated tRNA-Thr. Consistent with the idea that threonyl-tRNA is limiting during growth on fructose, threonyl-tRNA synthetase was

downregulated under lithotrophic conditions (Supplementary Table S13). The gene on the 5' side of the T-box (CLJU_RS03515), encoding an ACT domain protein of unknown function, was also downregulated (Supplementary Table S13); ACT domains often sense amino acids.

Threonine is a precursor of isoleucine and cobamide. There were no indications of increased isoleucine biosynthesis during growth with fructose (Supplementary Table S1), but the genes for enzymes that activate threonine for cobamide biosynthesis were downregulated for lithotrophic growth (Supplementary Table S13).

To explore the idea that *C. ljungdahlii* might need to synthesize proteins with high threonine content to grow organotrophically, the threonine content of every predicted protein of *C. ljungdahlii* was computed. Of those whose threonine content is at least 8.6%, which is two standard deviations (1.9%) above the mean (4.8%), most were not downregulated (data not shown). High-threonine-content protein genes that were downregulated with both syngas and H_2/CO_2 (Supplementary Table S14) encode a purine base transport membrane protein (CLJU_RS04170), a methyl-accepting chemotaxis protein (CLJU_RS06610), cell surface proteins (CLJU_RS21305, CLJU_RS21310, CLJU_RS21325), a microcompartment shell protein (CLJU_RS08810), and phage-specific genes (CLJU_RS15335, CLJU_RS15370, CLJU_RS15410, CLJU_RS15415, CLJU_RS15440, CLJU_RS15445). The cell surface protein genes are part of a cluster of genes that also encodes transporters for flavins and cobalt (Supplementary Table S15), interspersed with adenosylcobamide-responsive riboswitches (some of which are transcribed with CO/CO_2[2]). Several of these genes were downregulated with syngas and with H_2/CO_2. These genes are candidates for investigation of electron transfer through the cell wall, although the significance for growth with fructose is not obvious.

1. Nagarajan, H. *et al.* Characterizing acetogenic metabolism using a genome-scale metabolic reconstruction of *Clostridium ljungdahlii*. *Microb. Cell Fact.* **12,** 118 (2013).
2. Tan, Y., Liu, J., Chen, X., Zheng, H. & Li, F. RNA-seq-based comparative transcriptome analysis of the syngas-utilizing bacterium *Clostridium ljungdahlii* DSM 13528 grown autotrophically and heterotrophically. *Mol. Biosyst.* **9,** 2775–84 (2013).

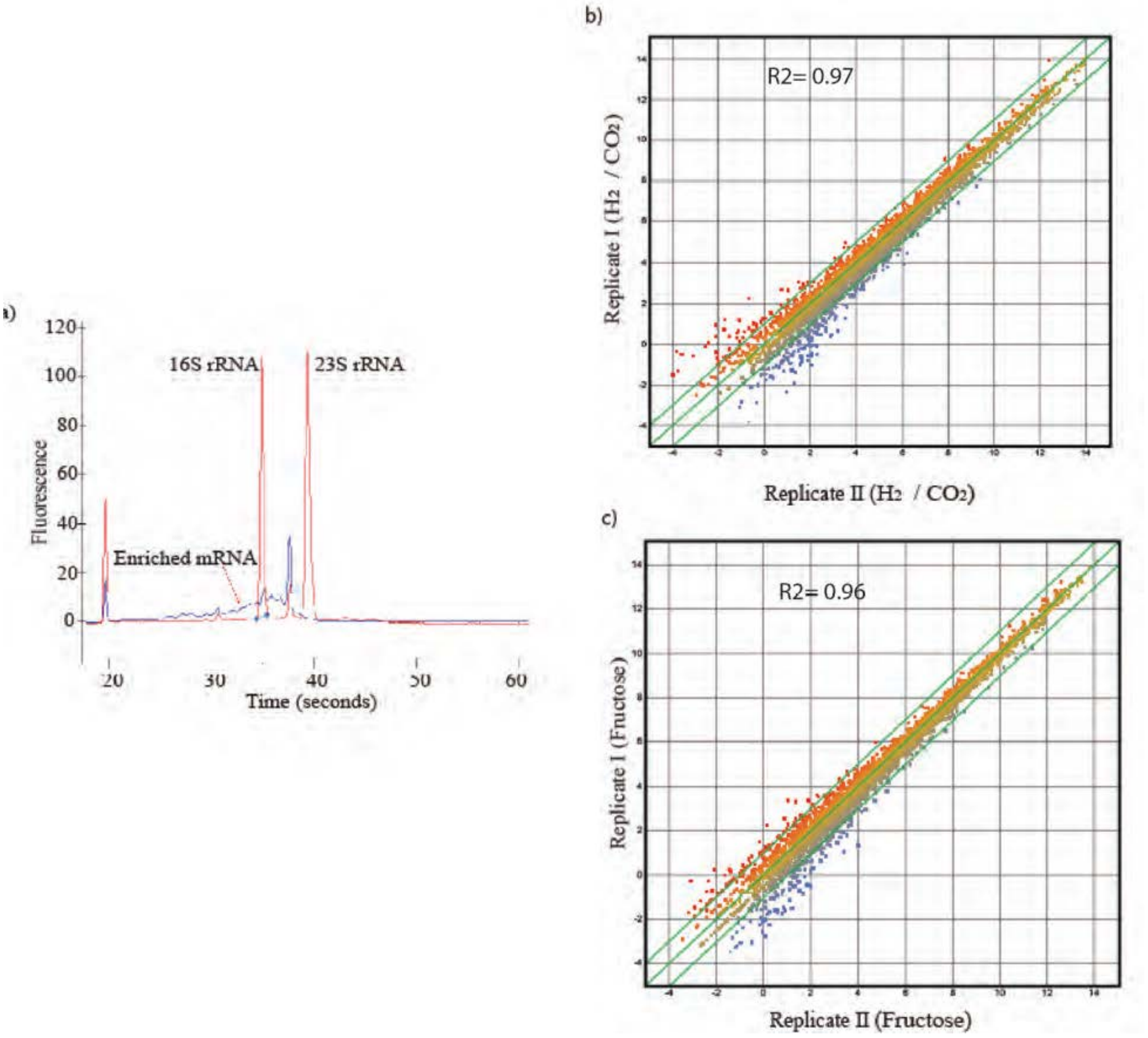

<u>Supplementary Figure S1.</u>

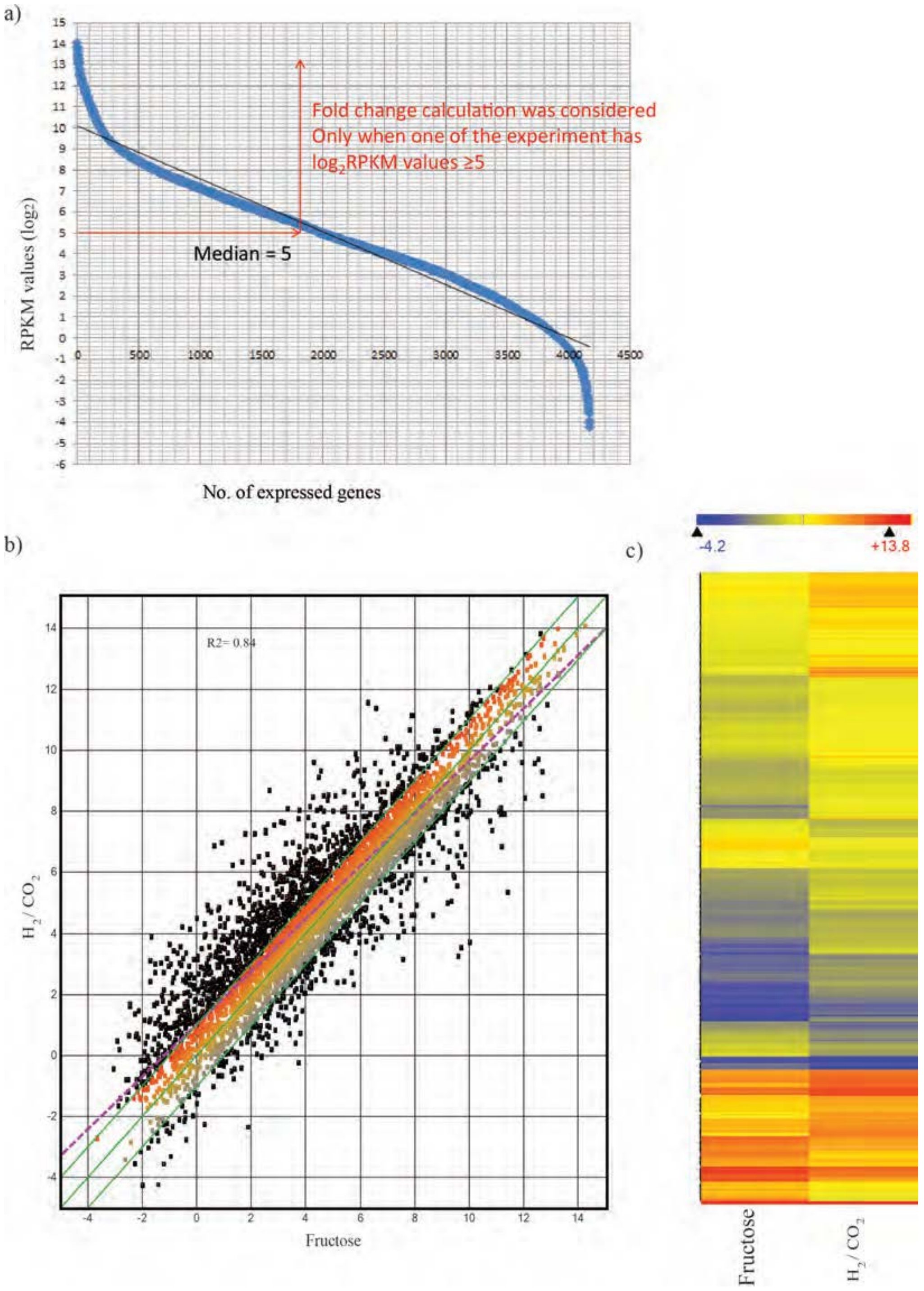

<u>Supplementary Figure S2.</u>
